# Time-Aware and Power-Law Retention Gating Mechanisms in LSTMs for Irregularly Sampled Sensor Data: A Survey

**DOI:** 10.3390/s26154868

**Published:** 2026-08-02

**Authors:** Diba Das, Scott D. Adams, Dean M. Corva, Tracey K. Bucknall, Abbas Z. Kouzani

**Affiliations:** 1 School of Engineering, Deakin University, Geelong, VIC 3216, Australia; d.das@deakin.edu.au (D.D.); scott.adams@deakin.edu.au (S.D.A.); 2 School of Engineering, Deakin University, Burwood, Melbourne, VIC 3125, Australia; d.corva@deakin.edu.au; 3 School of Nursing & Midwifery, Centre for Quality and Patient Safety Research, Institute for Health Transformation, Deakin University, Geelong, VIC 3220, Australia; tracey.bucknall@deakin.edu.au; 4 Alfred Care Group, Bayside Health, 55 Commercial Rd, Melbourne, VIC 3004, Australia

**Keywords:** Long Short-Term Memory, forget gate, irregularly sampled sensor data, time-aware recurrent networks, power-law decay, IoT time-series modelling

## Abstract

Sensor data streams from Internet of Things devices, wearables, and monitoring systems are often irregularly sampled due to variable transmission intervals, intermittent connectivity, and event-driven reporting, producing heavy-tailed temporal gaps between observations. Long Short-Term Memory (LSTM) networks, widely used for sequential sensor data analysis, assume a synchronous, equidistant timeline and regulate memory through a forget gate whose dynamics are input- and state-dependent but carry no explicit dependence on elapsed real time. Under stationary inputs, the standard LSTM architecture additionally exhibits a structural bias toward exponential memory decay. However, in real-world sensing contexts, uneven sampling and long-range dependencies are common, violating both assumptions simultaneously. To address this, we survey and taxonomise two independently proposed strategies: time-aware mechanisms that rescale memory using elapsed time, and power-law retention schemes that model slower, heavy-tailed forgetting. Through comparative analysis, we demonstrate that both strategies converge on the same underlying failure point, the inability of the forget gate to jointly adapt its attenuation rate and decay profile to temporal context and sensor input, and frequently employ functionally redundant mechanisms. We subsequently articulate a unified design framework that defines the essential properties that temporal forget gates must satisfy to enable robust learning from irregular sensor streams. These findings indicate that future sequence models for sensing applications should jointly integrate temporal-gap awareness with adaptive retention dynamics to better capture complex temporal dependencies in real-world environments.

## 1. Introduction

Artificial intelligence (AI) and deep learning (DL) have fundamentally reshaped how we analyse sequential data, providing the tools to extract complex patterns from continuous information streams. While these models have achieved remarkable success by learning temporal dynamics, their core architectures are typically optimised for data progressing along a uniform, discrete timeline [1,2]. In the domain of recurrent architectures, the Long Short-Term Memory (LSTM) network emerged as a landmark innovation, specifically engineered to alleviate the vanishing and exploding gradient problems inherent in standard Recurrent Neural Networks (RNNs) [3,4]. By introducing a specialised cell state regulated by multiplicative gating mechanisms, the LSTM enabled gradients to propagate across much longer temporal horizons, improving the modelling of long-range dependencies and making it a foundational architecture for sequence learning in industrial sensing applications [5,6,7].

**Figure 1:**
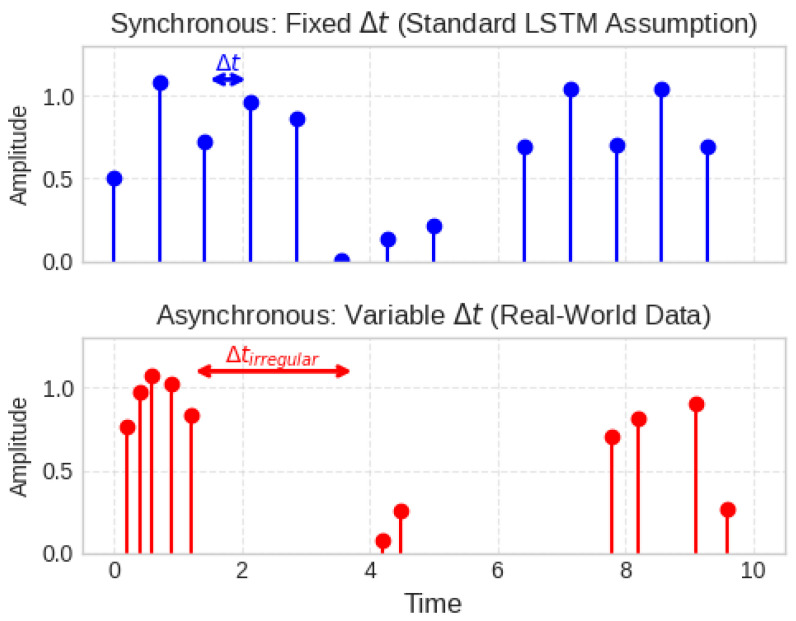
Illustration of the structural mismatch between uniform and asynchronous data.

Despite its success, LSTM’s foundational design relies on an assumption of synchronous uniformity, where time steps are equidistant. However, many real-world measurements do not follow sequential time steps, from sparse Electronic Health Records (EHRs) and bursty financial transactions to asynchronous industrial sensor arrays, and are inherently irregular, intermittent, and multi-frequency [8,9,10], as illustrated in Figure 1. When forced to process such unevenly spaced sequences, the standard LSTM approach faces a structural mismatch rooted in two primary architectural vulnerabilities. First, the network lacks a native mathematical mechanism to scale its internal state dynamics based on variable time gaps [11,12]. Second, because the standard LSTM’s forget gate applies a sigmoidal fraction recursively at every discrete step, the architecture is structurally biased toward exponential decay bounds over long horizons [13]. While a system’s physical dynamics do not strictly dictate the mathematical form of a predictive model’s internal memory kernel, this exponential bias can introduce learning inefficiencies when heavy-tailed retentions are expected. These are not merely theoretical concerns; in practice, they limit the ability of deployed LSTM models to preserve relevant historical states across the irregular, bursty observation patterns characteristic of real industrial sensor networks. Similar mismatches have been observed in sparse Electronic Health Records [14,15] and asynchronous financial transaction streams [16,17], confirming that the equidistant-step assumption and fast forgetting are foundational constraints rather than incidental limitations.

To address the first vulnerability, the research community has proposed a class of time-aware successors, including Time-Aware LSTM (T-LSTM) [14], Phased LSTM [18], etc., each addressing the issue from a different architectural angle. To address the second vulnerability, Power-Law-Based LSTM (pLSTM) [13] replaces the conventional forget gate with a learnable power-law decay, allowing the network to retain a heavy-tailed memory trace. Critically, these gate-level modifications address only a specific subset of the broader challenges posed by irregular sensor data. Other distinct issues require fundamentally different architectural paradigms, such as informative missingness, where missing channel patterns often carry predictive signals that models such as GRU-D can explicitly exploit [19]; asynchronous multi-rate alignment, where unsynchronised sensor clocks can be handled via upstream interpolation using interpolation–prediction networks [20] or non-recurrent continuous-time attention, such as Multi-Time Attention Networks (mTANs) [21] and Continuous-Time Transformer (ContiFormer) [22]; and trajectory fidelity vs. latency, where fully continuous formulations, such as Ordinary Differential Equations (ODEs)–RNNs [23,24], and Neural-Controlled Differential Equations (Neural CDEs) [25] trade deterministic, closed-form execution for solver-based trajectories, a trade-off that introduces unpredictable inference latency in edge-deployed industrial monitoring.

To delineate the scope of this work, this survey adopts a narrative review approach to synthesise and thematically organise gate-level modifications in the LSTM architecture designed for non-uniform sequential data. Rather than following a protocol-based systematic framework for a single use case, it establishes a cohesive taxonomy of structural modifications that directly address the dual limitations of temporal insensitivity and rigid decay. To construct this overview, the search initiated with landmark papers on Time-Aware LSTMs, utilising both forward and backward citation tracking, and subsequently expanded to encompass Power-Law LSTM architectures for long-range dependencies. Articles were included only if they provided distinct theoretical insights or key empirical advancements regarding gate modifications for irregularly sampled sensor data, while highly localised case studies and preliminary reports were excluded. Ultimately, this comprehensive synthesis serves as a practical reference for researchers and engineers designing robust sequence models tailored for real-world industrial sensing environments.

The key contributions of this survey are threefold:

1. We present a unified taxonomy of time-aware LSTMs and power-law retention models for irregularly sampled sequences.
2. We show that these approaches address a common limitation of standard forget gates through functionally overlapping mechanisms.
3. We articulate a conceptual framework that identifies the essential properties of adaptive temporal forget gates and outlines directions for future research.

The remainder of this survey is structured as follows. Section 2 establishes the foundational mathematical formulation of the LSTM and the limitations of sigmoidal forget gates. Section 3 presents a taxonomy of LSTM variants designed for irregular time steps. Section 4 analyses heavy-tailed memory retention using the power-law gate in LSTM. Section 5 first proposes a unified conceptual framework synthesising the essential properties of adaptive temporal forget gates and then provides discussion on competing continuous-time and attention-based paradigms, concluding with practical training, hyperparameters, and deployment considerations. Finally, Section 6 concludes this paper.

## 2. Background

**Figure 2:**
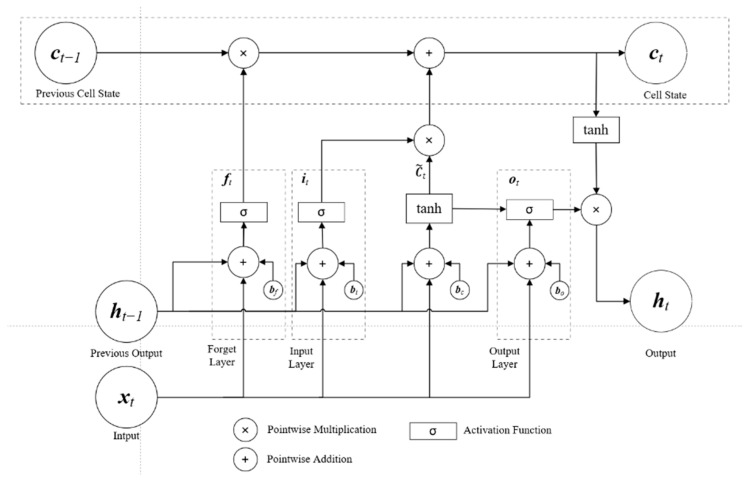
The cell structure of the LSTM.

RNNs maintain an internal state by combining current inputs with past information. Standard RNNs often struggle with long-term dependencies due to vanishing gradients; an LSTM network [3] mitigates this with a gating framework and a specialised cell state ($C_{t}$) that facilitates stable gradient flow. The LSTM architecture, as shown in Figure 2, regulates information flow through three learned mechanisms: the input gate ($i_{t}$), forget gate ($f_{t}$) and output gate ($O_{t}$). Formally, for an input $x_{t}$ and the previous hidden state $h_{t-1}$, the cell dynamics are governed by the following standard formulation [3,13]:

(1) $$i_{t}=\sigma (x_{t}W_{ix}+h_{t-1}W_{ih}+b_{i})$$

where $W_{ix}$ and $W_{ih}$ are the weight metrics, and $b_{i}$ is the bias vector.

(2) $$f_{t}=\sigma (x_{t}W_{fx}+h_{t-1}W_{fh}+b_{f})$$

where $W_{fx}$ and $W_{fh}$ are the weight metrics, and $b_{f}$ is the bias vector.

(3) $$o_{t}=\sigma (x_{t}W_{ox}+h_{t-1}W_{oh}+b_{o})$$

where $W_{ox}$ and $W_{oh}$ are the weight metrics, and $b_{o}$ is the bias vector.

The updates to the cell state and hidden state are governed by the following equations:

(4) $${\tilde{C}}_{t}=\tanh\left(x_{t}W_{cx}+h_{t-1}W_{ch}+b_{c}\right)$$

where $\tilde{C}$ is the candidate cell state, $W_{cx}$ and $W_{ch}$ are the weight metrics, and $b_{c}$ is the bias vector. Then,

(5) $$C_{t}=f_{t}⊙C_{t-1}+i_{t}⊙{\tilde{C}}_{t}$$

(6) $$h_{t}=o_{t}⊙\tanh\left(C_{t}\right)$$

Because these equations omit elapsed time, the network treats observations as occurring at discrete, equidistant intervals. Consequently, the LSTM cannot distinguish between dense observation clusters and sparse, asynchronous data. While architectural variants like the Gated Recurrent Unit (GRU) [26,27], the Minimal Gated Unit (MGU) [28] and the Gated Orthogonal Recurrent Unit (GORU) [29] improve parameter efficiency or gradient stability, they maintain the baseline assumption of temporal uniformity. Their single-channel designs struggle to incorporate continuous time-decay signals without corrupting stored historical context. Thus, addressing irregular timelines and slowing down memory decay requires moving beyond these parameter-centric modifications toward explicit time-aware gating, as detailed in the following sections.

## 3. Taxonomy of Time-Aware Architectures

The limitations of the standard LSTM under irregular sampling have motivated a diverse family of architectural responses, each targeting the core equidistant-step assumption from a different angle. These responses broadly cluster around five design strategies. The first modifies the forget gate directly to make memory decay an explicit function of elapsed time ∆t, as seen in DeepCare [30], treating forgetting as a temporal process rather than a fixed recursive operation. The second decomposes the cell memory into separable long-term and short-term subspaces before applying time-dependent discounting selectively, as in T-LSTM [14], preserving stable long-range context while allowing recent information to decay appropriately. The third introduces dedicated time gates as independent architectural components that regulate how strongly temporal distance influences information flow at the current step, as in Time-LSTM [31]. A closely related variant is Multi-Way Adaptive Time-Aware (MWTA)–LSTM [15], whose bottom layer is built upon an extended modified version of T-LSTM and Time-LSTM. The fourth strategy, represented by Attention-Based Time-Aware LSTM (ATTAIN) [32], takes a retrospective approach: rather than modifying how the current state is computed from the immediately preceding step, it accumulates cell states across all previous events and reweights them jointly using learned attention scores for clinical salience alongside a continuous-time decay function that penalises temporally distant events. A fifth strategy, embodied by Phased LSTM [18], decouples network updates from data arrival entirely, treating time as a gating coordinator that determines when information is allowed to flow, rather than applying a decay multiplier to what has already flowed. While these approaches differ in mathematical formulation and intended domain, they share a common motivation: encoding elapsed time as a signal within the recurrent architecture, rather than leaving the network to infer temporal structure implicitly from sequential position alone. The following subsections are organised by design strategy rather than chronological order of publication, reflecting the distinct architectural philosophies each approach represents.

### 3.1. DeepCare

Standard LSTM forget gates apply a fixed sigmoidal decay at every step, regardless of how much real time has elapsed between admissions. DeepCare [30] was among the first architectures to address this directly in a clinical context, augmenting the LSTM cell by making the forget gate an explicit function of the irregular time gap ∆t between consecutive hospital admissions. It introduces two distinct forgetting behaviours depending on modelling assumptions: a monotonic time decay for settings where memory should fade smoothly and predictably, and a fully parametric time-aware forget gate for settings where interventions and care processes must also be encoded.

#### 3.1.1. Mode 1: Monotonic Time Decay

Where clinical memory is assumed to fade smoothly with elapsed time, the forget gate is first computed normally and then scaled by a logarithmic decay factor [30].

(7) $$d(∆t)={\left[\log\left(e+∆t\right)\right]}^{-1}$$

(8) $$f_{t}\leftarrow d(∆t).f_{t}$$

Here, ∆t is the elapsed time between admissions at *t* and *t* − 1, and d(∆t) is a monotonically decreasing function that approaches zero as the interval grows. This discounts the carry-forward of older cell memory proportionally to the length of the gap.

#### 3.1.2. Mode 2: Forgetting Through Parametric Time

Where the time gap and clinical interventions carry informational weight of their own, the forget gate is fully re-parameterised to ingest them directly:

(9) $$f_{t}=\sigma (x_{t}W_{fx}+h_{t-1}W_{fh}+q_{∆t-1:t}W_{fq}+p_{t-1}W_{fP}+b_{f})$$

where $q_{∆t-1:t}$ is a learned time embedding encoding the elapsed interval, and $p_{t-1}$ is an intervention vector capturing the care process applied in the preceding step. This allows the gate to learn not just that time has passed, but what kind of clinical activity occurred in that interval. Together, these two modes allow DeepCare to treat forgetting as a temporally grounded process rather than a uniform recursive operation. However, both modes apply time discounting to the entire cell state without distinguishing between information that should decay and information that should persist, a limitation directly addressed by the architecture of T-LSTM.

### 3.2. Time-Aware LSTM

Applying time decay to the full cell state indiscriminately risks discounting stable, long-term signals alongside genuinely transient ones. To overcome this, Baytas et al. [14] proposed the Time-Aware LSTM (T-LSTM), which decomposes the previous cell memory $C_{t-1}$ into distinct long-term and short-term subspaces before applying any time discounting. Only the short-term component is subject to temporal decay, leaving long-range contextual memory structurally intact.

The decomposition and discounting proceed in four steps. First, the short-term memory subspace is extracted via a learned projection [14]:

(10) $$C_{t-1}^{S}=\tanh\left(C_{t-1}W_{d}+b_{d}\right)$$

where $W_{d}$ and $b_{d}$ are trainable parameters defining the subspace. The short-term component is then discounted according to the elapsed interval using a non-increasing decay function g(∆t):

(11) $${\hat{C}}_{t-1}^{S}=C_{t-1}^{S}*g(∆t)$$

The decay function g(∆t) is selected based on the temporal scale of the dataset:

g(∆t)=1/∆t for datasets with a small amount of elapsed times.

$g(∆t)={\left[\log\left(e+∆t\right)\right]}^{-1}$ for datasets with large elapsed times.

where Δ*t* is the time span between records at step *t* and *t* − 1.

The long-term component is recovered as the remainder of the original cell state after the short-term subspace is removed:

(12) $$C_{t-1}^{T}={C_{t-1}-C}_{t-1}^{S}$$

These two components are then recombined to form the adjusted memory that enters the standard LSTM gating computation:

(13) $$C_{t-1}^{*}=C_{t-1}^{T}+{\hat{C}}_{t-1}^{S}$$

The resulting $C_{t-1}^{*}$ preserves stable long-range dependencies in full while selectively discounting recent, transient information proportionally to the elapsed gap. This makes T-LSTM particularly effective for longitudinal patient records where early diagnostic history must coexist with recent clinical events. However, T-LSTM still derives each cell state update solely from the immediately preceding step; it has no mechanism to attend retrospectively across the full history of past states, a capability introduced in Section 3.4.

### 3.3. Time-LSTM

Both DeepCare and T-LSTM treat time as a corrective modifier applied to a cell state that has already been formed through standard gating operations. Time-LSTM [31] takes a structurally different position: rather than scaling what has already been computed, it embeds the elapsed time interval ∆t directly inside dedicated time gates that participate in the cell state computation itself. This distinction matters because it allows temporal distance to govern not just how much past context survives, but how new information is written into memory in the first place.

A critical distinction this paper draws is between modelling the timestamp of a single event and modelling the time interval between two consecutive events. Phased LSTM [18], the closest prior architecture, operates on the former, using the absolute timestamp as the input to its time gate. Zhu et al. [31] argue that this is insufficient for recommendation settings on three grounds. First, it may fail to properly model the relational significance between two actions, since what matters behaviourally is not when an action occurred in absolute terms, but how long after the previous one occurred. Second, because Phased LSTM enters an inactive state during long gaps and ignores data points that fall outside its oscillation window, it discards useful behavioural signals in the sparse action sequences typical of recommender systems. Third, and most fundamentally, it provides no architectural mechanism to separately capture and jointly balance two qualitatively distinct interest signals that the prior recommendation literature identifies as critical: (i) short-term interest, the immediate contextual dependency between recent consecutive actions, for example, a user who purchases a camera is highly likely to purchase a memory card or lens in the near future; (ii) long-term interest, the stable, persistent preference profile of a user that shapes all recommendations regardless of recency, the general behavioural tendency that persists across months or years of interaction history. Standard LSTM and Phased LSTM conflate these two signals within a single hidden state. Time-LSTM architecturally separates them through independently parameterised time gates. Time-LSTM exists in three versions, each extending the previous with greater temporal expressiveness. The simplest version adds a one-time gate $T_{m}$ that takes $\Delta t_{m}$ as an explicit input alongside the current item embedding [31]:

(14) $$T_{m}=\sigma_{t}(x_{m}W_{xt}+\sigma_{\Delta t}(\Delta t_{m}W_{tt})+b_{t})$$

where $x_{m}$ is the input feature vector of the mth item, $\Delta t_{m}$ is the elapsed time since the previous action, and $\sigma_{t}$ denotes a sigmoid applied to the time-scaled term. The second version uses ${T1}_{m}$ and ${T2}_{m}$:

(15) $${T1}_{m}=\sigma_{1}(x_{m}W_{x1}+\sigma_{\Delta t}(\Delta t_{m}W_{t1})+b_{1})s.t.\;W_{t1}\leq 0$$

(16) $${T2}_{m}=\sigma_{2}(x_{m}W_{x2}+\sigma_{\Delta t}(\Delta t_{m}W_{t2})+b_{2})$$

${T1}_{m}$ retains its recency-sensitive, monotonically decreasing behaviour and is responsible for capturing the short-term interest. ${T2}_{m}$ can learn an unconstrained relationship between the elapsed interval and the long-term preference signal, allowing it to represent stable interest contexts that are not necessarily sensitive to how much time has passed. The two gates modulate separate pathways within the cell state update, giving the architecture independent control over each interest type for the first time. The third version preserves the dual-gate temporal structure but replaces the separate input and forget gates with coupled gates. Despite this expressiveness across all three versions, Time-LSTM produces no mechanism for attributing predictions back to specific past events in the sequence history. Each cell state update is still derived solely from the immediately preceding step, a structural constraint that becomes a significant limitation in high-accountability domains such as clinical forecasting, where the basis of a prediction must be traceable and explainable. This gap is directly addressed by the architecture in Section 3.5.

### 3.4. Multi-Way Adaptive Time-Aware LSTM

Multi-Way Adaptive Time-Aware LSTM (MWTA-LSTM) [15] addresses time and frequency issues. It introduces two stacked recurrent units, each contributing a distinct decay signal, whose outputs are fused through multi-way spatial–temporal pooling. The first unit extends a T-LSTM base with a dedicated time gate $T_{t}$ that conditions the cell state update directly on the elapsed interval [15]:

(17) $$T_{t}=\sigma (W_{x_{t}}x_{t}+\sigma_{\Delta_{t}}(g(\Delta_{t}))+b_{t})$$

where $g(\Delta_{t})$ is a non-increasing function of the elapsed time $\Delta_{t}$, and σ denotes a sigmoid applied to the time-scaled term. This gate regulates how much of the new input influences the cell state as a function of elapsed time. The second unit augments the forget gate with a combined decay term that accounts for both temporal distance and sampling frequency:

(18) $$f_{t}=\overset{Time\text{-}based\;\mathrm{decay}}{\overbrace{f_{t}*t_{d}}}+\overset{Frequency\text{-}based\;\mathrm{decay}}{\overbrace{(1-f_{t})*f_{d}}}$$

where $t_{d}$ is a time-based decay scalar, and $f_{d}$ is a frequency-based decay scalar, both learned from training data. The term $(1-f_{t})*f_{d}$ acts as a complementary signal: when the standard forget gate $f_{t}$ is small (indicating recent, relevant content should be retained), the frequency decay term contributes proportionally more, preventing sudden sampling bursts from artificially amplifying or suppressing cell-state content. Together, the two units insulate long-term hidden state representations from outlier sampling frequencies without discarding the temporal sensitivity established in Unit 1. Nevertheless, both units still process every incoming data point regardless of the elapsed interval; updates are triggered by data arrival, not by temporal significance. This coupling of update frequency to data arrival is the structural assumption that the architecture in Section 3.6 explicitly breaks.

### 3.5. Attention-Based Time-Aware LSTM

DeepCare, T-LSTM, and Time-LSTM all share a structural constraint: each cell state $C_{t}$ is derived strictly from the immediately preceding state $C_{t-1}$. Rather than passing only $C_{t-1}$ into the current update, Attention-Based Time-Aware LSTM Networks (ATTAINs) [32] accumulate cell states from every previous event and compute a combined memory weighted by two independent sources:

(19) $$C_{t-1}^{*}=\sum_{i}W_{i}\cdot C_{i}$$

where each combined weight $W_{i}$ is defined as

(20) $$W_{i}=\alpha_{i}\cdot \delta (t-t_{i})$$

Here, $\alpha_{i}$ is a learned attention weight reflecting the clinical salience of event *i*, derived from the hidden state context at that step, and $\delta (t-t_{i})\;$ is a continuous-time decay function that penalises events proportionally to their temporal distance from the current prediction point. Events that are both clinically salient and temporally proximate, therefore, receive the highest combined weight, while events that are distant or contextually irrelevant are down-weighted along both dimensions simultaneously. This structure produces a natively interpretable output: the weights $W_{i}$ constitute a human-readable attribution map showing exactly which past events and time intervals drove each prediction. This makes ATTAIN particularly effective in accountability-sensitive settings, such as septic shock forecasting, where clinicians require not just a prediction but a traceable explanation. The cost of this retrospective design is increased computational load relative to single-step architectures. Additionally, ATTAIN does not explicitly address erratic or multi-frequency sampling noise, a challenge that arises acutely in ICU environments and that the architecture in Section 3.5 is specifically designed to handle.

### 3.6. Phased LSTM

All architectures discussed so far operate as passive decay models; they accept every incoming data point and apply time-dependent modulation after the fact. Phased LSTM [18] takes a structurally different position: rather than modulating what is remembered after each update, it controls whether an update occurs at all, decoupling network state transitions from raw data arrival. It introduces a new time gate $k_{t}$ that operates alongside the standard input, forget, and output gates. $k_{t}$ is governed by a parametric oscillation defined by three learnable parameters: a period τ, a phase shift *s*, and an open ratio $r_{on}$ that determines what fraction of each cycle the gate remains open. When the gate is closed, the cell state and hidden state pass through unchanged, and the network effectively skips the step; when open, the update proceeds normally. Because the open ratio is typically set low, most steps are skipped entirely, which, in the original formulation, also yields substantial computational savings alongside faster convergence on long sequences. This structure is naturally suited to asynchronous multi-sensor streams, where different sensors fire at entirely independent rates. Each sensor’s Phased LSTM unit learns its own oscillation period independently, allowing the architecture to synchronise internal updates to the semantically meaningful rhythm of each input channel rather than to the raw data clock. By treating time as a coordinator for when information flows, rather than as a multiplier applied to what already exists, this architecture represents a qualitatively different design philosophy from the decay-based and attention-based families covered in Section 3.1, Section 3.2, Section 3.3, Section 3.4 and Section 3.5.

## 4. Taxonomy of Power-Law Retention Models

The standard LSTM offers no mechanism for representing heavier-tailed dependency structures. For sequences with heavy-tailed dependency structures or irregular temporal sampling, retention may take several functional forms; this section focuses specifically on power-law models.

### 4.1. Power-Law-Based LSTM

The Power-Law-Based LSTM (pLSTM) [13] is a modification of the standard LSTM to better model long-term dependencies in irregular temporal data. The key idea is to replace or augment the standard memory decay of the LSTM with a power-law memory mechanism, allowing information to fade more gradually over time. Rather than exponential decay, pLSTM proposes a forget gate based on the power-law function [13]:

(21) $$f_{t}={\left(\frac{t-k_{t}+1}{t-k_{t}+\epsilon }\right)}^{-p}$$

where $k_{t}\;$ is the reference time. The power p is the key parameter. The power-law decay is slower as p  approaches 0, suggesting that long-range dependencies are better captured with small values of p. The power p is treated as a learnable parameter. A small ε = 0.001 is added to the denominator to prevent numerical instability during backpropagation. This gate grants a slower information decay rate. The complete gating mechanism is defined as

(22) $$k_{t}=r_{t}⊙t+(1-r_{t})⊙k_{t-1}$$

(23) $$r_{t}=\sigma (U_{r}x_{t}+W_{r}h_{t-1}+b_{r})$$

(24) $$c_{t}=f_{t}⊙c_{t-1}+i_{t}⊙{\tilde{c}}_{t}$$

where a reset gate $r_{t}\;$ controls when the reference time $k_{t}\;$ should be updated based on the input and recurrent information. When the reset gate is activated ($r_{t}$ → 1), the reference time resets to the current timestep *t*; otherwise, $k_{t}\;$ is held at its prior value to allow information to continue decaying from that reference point.

### 4.2. Time-Aware LSTM with Power-Law Decay

Kim [33] developed a Time-Aware with Power-Law Decay LSTM (T-pLSTM), a direct hybrid of T-LSTM and pLSTM. It retains T-LSTM’s adjusted memory cell to handle irregular time intervals, and replaces the standard forget gate with pLSTM’s power-law forget gate to handle long-timescale retention. T-pLSTM can handle long-timescale patient records with irregular elapsed time by a power-law forget gate and an adjusted memory cell. The adjusted memory cell (inherited from T-LSTM) decomposes the previous cell state into short-term and long-term subspaces, discounting only the short-term component by a function of elapsed time ∆t, preserving stable long-range clinical history while allowing recent transient signals to decay appropriately. T-pLSTM is the only architecture in the literature that simultaneously uses time as a decay scalar (the adjusted memory cell from T-LSTM) and non-exponential retention (the power-law forget gate from pLSTM). It is not a new design philosophy; rather, it is a principled hybridisation of two existing approaches that individually addressed only one of the two failure modes. Kim [33] also introduces a bidirectional version, Bidirectional Time-Aware with Power-Law Decay LSTM (BiT-pLSTM), which processes the patient sequence in both forward and backward directions simultaneously. Training in two directions, exploited in Bi-LSTM, often leads to better performance than traditional LSTM. The bidirectional extension allows the model to incorporate both past clinical history and future trajectory context when making predictions at any given timestep, particularly useful in oncology, where both prior progression and eventual outcomes are informative.

## 5. Discussion and Future Directions

The structural adaptations detailed in this section represent cell-level engineering designed to adapt discrete-time recurrence to more realistic real-world continuous dynamics. However, the modern sequential modelling landscape is defined by a broader architectural debate: whether temporal non-uniformity should be managed via analytical, discrete-time gating extensions or delegated to entirely continuous-time differential equations and linear state-space formulations.

### 5.1. Towards a Unified Temporal Gating Framework

The survey of architectures in Section 3 and Section 4 reveals a recurring structural pattern across time-aware and power-law retention models. Each design independently compensates for the same root limitation of the standard forget gate, namely, its inability to jointly adapt its attenuation speed and decay curve shape as a function of elapsed time and input content. In T-pLSTM specifically, this pattern produces visible redundancy: the subspace decomposition, the monotone short-term decay correction, and the power-law gate each address a different symptom of the same underlying cause, resulting in a three-stage pipeline whose complexity is not justified by a three-way distinction in the problem.

The claim that time-aware and power-law models compensate for the same underlying limitation is demonstrated below by decomposing each architecture into the elementary mechanisms it uses. Three deficiencies of the standard forget gate motivate every architecture reviewed in Section 3 and Section 4: (P1) insensitivity to elapsed time Δt, (P2) lack of heavy-tailed retention, and (P3) a fixed, non-adaptive parameterisation of the decay rate and shape. Table 1 classifies each model by which of the six recurring elementary mechanisms it uses to address these deficiencies.

DeepCare, T-LSTM, Time-LSTM, MWTA-LSTM, ATTAIN, and Phased LSTM each address P1 alone, but do so through five structurally distinct mechanisms (scalar gate scaling, explicit gate embedding, subspace-limited discounting, attention-weighted decay, and oscillatory gating), none of which address P2 or P3. Only pLSTM addresses P2 alone via a single substituted gate function. T-pLSTM and BiT-pLSTM are the only architectures that combine mechanisms across columns, and they do so by concatenating two already-existing solutions (T-LSTM’s subspace decomposition and pLSTM’s power-law gate) rather than by deriving a new mechanism that satisfies both properties jointly. No model in the taxonomy addresses P3. This mechanism-level view supports three conclusions. First, mechanisms used to address P1 are functionally interchangeable at the level of the property they solve, which is evidence of redundant parallel invention rather than principled architectural diversity. Second, T-pLSTM and BiT-pLSTM’s combination of subspace decomposition and power-law gating is additive rather than integrative: it stacks two P1/P2 solutions side by side instead of producing a mechanism that satisfies both from a shared parameterisation. Third, the lack of addressing P3 is direct evidence for the gap: no existing mechanism, however it is combined with others, makes decay simultaneously time-aware, non-exponential, and content-adaptive.

The pattern observed suggests a broader design principle worth articulating rather than assembling independent corrective modules in sequence: a more parsimonious architecture would integrate temporal sensitivity, decay rate control, and curve shape into a single, jointly learned gating operation, in which the forget gate receives elapsed time and input content as first-class inputs and determines both the speed and shape of decay as data-dependent functions, eliminating the need for a pre-processing decomposition stage. This design principle can be stated as three functional properties a unified temporal forget gate should satisfy jointly: sensitivity to elapsed time (P1), heavy-tailed decay (P2), and content-aware adaptation of both the decay rate and decay shape (P3).

**Figure 3:**
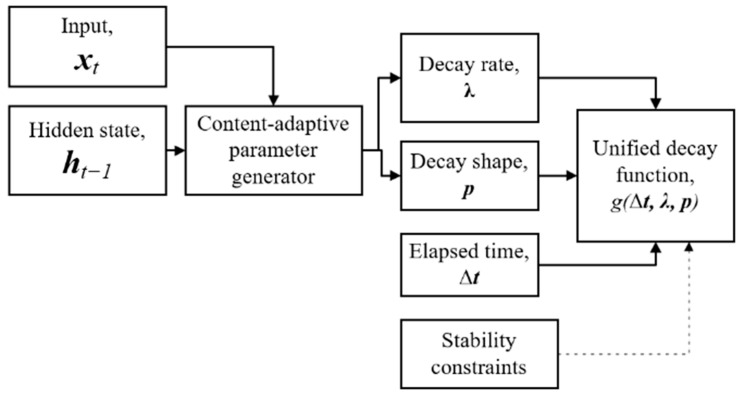
An exemplary framework for the unified gate. Solid arrows denote data dependencies; the dashed arrow denotes the stability constraints (boundedness, monotonicity in Δt, and the Δt = 0 boundary condition) that the unified decay function g must satisfy.

To move beyond stating these properties qualitatively, we propose an exemplary framework, as given in Figure 3. We provide candidate parameterisation and verify analytically that it satisfies all three considered principles.

Let a generator produce decay parameters directly from the current input and hidden state:

(25) $$\lambda (t)=\mathrm{softplus}(W_{\lambda }{[x}_{t};h_{t-1}]+b_{\lambda })$$

(26) $$p(t)=p_{min}+\sigma (W_{p}{[x}_{t};h_{t-1}]+b_{p})$$

and let these jointly parameterise a generalised power-law forget gate:

(27) $$g(∆t,\;\lambda ,\;p)=\frac{1}{{\left(1+\lambda (t)\cdot \Delta t\right)}^{p(t)}}$$

This formulation satisfies P1, since $\frac{\partial g}{\partial ∆t}=-\frac{p(t)\lambda (t)}{{\left(1+\lambda (t)\Delta t\right)}^{p(t)+1}}$ < 0 for all λ, p > 0, so retention decreases strictly monotonically with elapsed time. It satisfies P2, since g decays polynomially in ∆t. It satisfies P3 by construction, since λ(t) and p(t) are functions of x(t) and h(t−1) rather than fixed hyperparameters. Boundedness and stability constraints must be verified for this formulation to be well-posed. In this case, these follow directly from the same construction: because λ, p > 0, 1+λ(t)Δt≥1 for all Δt≥0, so g(∆t)∈(0,1]. The formulation above is presented as a candidate mechanism supported by analytical derivation of P1–P3 and the associated stability guarantee; it has not yet been empirically validated. This is the primary direction for future work: implementing the gate above as a drop-in replacement for the standard forget gate and benchmarking it against the architectures to determine whether the analytical gains in P1–P3 translate into measurable improvements in retention quality, predictive accuracy, and training stability, and whether the generator above requires additional capacity.

### 5.2. Competing Paradigms: Neural ODEs, State-Space Models, and Continuous-Time Attention vs. LSTMs

The primary alternatives to time-aware recurrent networks belong to two major architectural families: Neural Ordinary Differential Equations (Neural ODEs) [34] and Structured State-Space Models (SSMs). Both paradigms fundamentally reshape how history and continuous time are represented in sequence modelling. Neural ODEs parameterise the continuous derivative of the network’s hidden state trajectory using a neural network, bypassing discrete layer or step indices entirely. When an asynchronous observation arrives, a numerical solver integrates this continuous trajectory across the exact elapsed continuous time interval to advance the hidden state. Building on these continuous foundations, selective SSMs [35] map an input signal to a latent state, and then project it to an output via a linear first-order differential system. To process digitised sequences, these underlying equations are discretised using a time-step parameter. In models like Mamba [35], this parameter is calculated as a data-dependent, dynamic function of the input, enabling the architecture to act as a selective filter that scales its historical retention based on the precise temporal spacing and content of incoming elements. While these alternative frameworks are mathematically elegant, they introduce distinct system-level trade-offs when contrasted with structurally modified recurrent cells. First, neural ODEs rely on iterative numerical solvers that evaluate the trajectory multiple times per interval to satisfy strict local error tolerances. This introduces highly variable forward and backward pass latency [34]. In contrast, time-aware LSTMs compute time-decay adjustments analytically in a single step using closed-form expressions of the elapsed time, ensuring deterministic, fast, and highly predictable runtimes during training and edge deployment. Second, numerical ODE solvers remain vulnerable to error accumulation and gradient instability when sequences span thousands of steps [25].

Another competing paradigm dispenses with recurrent decay altogether in favour of attention over a continuous-time representation. Multi-Time Attention Networks (mTANs) [21] replace the forget gate with a learned continuous-time embedding, a direct generalisation of the transformer’s positional encoding to continuous time, paired with multi-head cross-attention that re-represents an irregular observation sequence at a fixed set of reference points. There is no decayed hidden state to characterise in the terms used elsewhere in this survey, because the mechanism does not propagate memory forward at all; it reconstructs a fixed-length representation from all observations directly. ContiFormer [22] goes further, embedding Neural ODE dynamics inside the attention mechanism itself, so that queries, keys, and values evolve continuously between observations. This directly extends the discrete-step assumption underlying standard Transformer attention into continuous time, at a computational cost markedly higher than either a standard Transformer or the recurrent architectures reviewed in Section 3 and Section 4, since it requires solving an ODE within every attention computation.

Conversely, structurally modified recurrent cells retain explicit, bounded gating layouts that stabilise gradient flow [36]. This enables simple, time-decaying LSTMs and GRUs to deliver highly competitive accuracy on irregular multivariate forecasting tasks without complex numerical solver loops. Also, selective SSMs exploit parallel associative scan algorithms to achieve sub-quadratic training speeds over massive contexts. However, this optimisation strictly requires a linear latent transition structure. While time-aware LSTMs scale sequentially, their fully non-linear gating loops allow them to learn intricate, state-dependent interactions where the rate of memory erasure adapts non-linearly to the structural importance of incoming data. Table 2 shows a summary of key comparisons of the LSTM variants discussed in this survey and other alternative architectures.

### 5.3. Practical Considerations: Training Difficulty, Hyperparameter Sensitivity, and Deployment Challenges

While asymptotic complexity characterises ideal efficiency, production deployment in Industrial Internet of Things (IIoT) settings depends on training stability, hyperparameter tuning burden, and edge hardware execution constraints. This subsection synthesises these practical dimensions across the architectures reviewed in Section 3, Section 4, Section 5.1 and Section 5.2.

Training difficulty generally increases with the number of interacting temporal mechanisms. Single-decay models such as DeepCare and T-LSTM can typically be optimised using standard first-order methods. Dual-gated architectures, including Time-LSTM and MWTA-LSTM, introduce larger hyperparameter search spaces that require balancing multiple temporal decay processes. Multi-mechanism variants, such as T-pLSTM and BiT-pLSTM, further increase complexity by combining subspace decomposition, short-term discounting, and power-law retention. ATTAIN incurs training time proportional to sequence length due to full-window attention weighting, whereas continuous-time models (Neural ODE/CDE) rely on numerical solvers or adjoint methods, where training stability hinges on non-standard solver tolerances. In case of hyperparameter sensitivity, discretely gated baselines rely on standard hidden-unit and learning-rate tuning. Adding a power-law coefficient expands this burden significantly; empirical findings reveal it varies nearly threefold across task regimes and sequence lengths, offering no universal default. Phased LSTM introduces initialisation sensitivity, where poorly chosen period or phase parameters can permanently desynchronise units from sensor streams. Neural ODEs/CDEs introduce solver choice alongside relative and absolute error tolerances, complicating standard tuning pipelines. For deployment and edge execution issues, discrete gate architectures compute temporal adjustments analytically in closed form, guaranteeing the predictable, bounded latency required for real-time edge monitoring. Phased LSTM further reduces compute by skipping inactive unit updates. Conversely, ATTAIN’s memory footprint grows linearly with sequence length, risking memory exhaustion in continuous monitoring. Neural ODE/CDE inference latency is inherently unpredictable due to variable solver step counts. Finally, state-space models like Mamba offer sub-quadratic scaling, but these gains rely on GPU-class parallel scan primitives that do not automatically translate into resource-constrained edge hardware lacking dedicated kernel support. Stacking independent correction mechanisms, whether temporal subspaces, power-law coefficients, or solver tolerances, compounds training difficulty and deployment overhead. This reinforces the need for unified, single-gate temporal operations.

## 6. Conclusions

This paper has addressed a fundamental challenge at the intersection of sequence modelling and Intelligent Industrial IoT: the structural mismatch between the synchronous design of Long Short-Term Memory (LSTM) networks and the irregularly sampled nature of industrial sensor streams. While standard LSTMs remain foundational for sequential analysis, their reliance on equidistant time steps and rigid memory decay limits their capacity to model long-term dependencies in the non-stationary environments characteristic of condition monitoring, fault detection, and predictive maintenance. Our survey demonstrates that existing architectures are partial instantiations of a deeper, unrealised design objective. The redundancy observed across these models, where independent mechanisms compensate for the same root cause, highlights the need for a more principled approach. We have articulated a unified design framework grounded in three essential properties: elapsed-time sensitivity, heavy-tailed retention, and content-aware parameterisation of decay rate and curve shape, each directly translatable into the operational demands of real IIoT sensing pipelines. The central contribution of this review is to shift the discourse from patching individual LSTM limitations toward formalising a unified temporal forget gate that collapses multi-stage pipelines into a single, differentiable operation, reducing deployment complexity in safety-critical industrial systems. How such a gate generalises across heterogeneous IIoT sensor modalities and scales within modern foundation models remains the principal direction for future research.

## Figures and Tables

**Table 1:** Summary of mechanism overlap.

(Bidirectional)	∆t-Scaled Forget Gate	∆t as Explicit Gate Input	Subspace Decomposition	Power-Law Gate	Retrospective Attention	Oscillatory Gating	Properties Addressed
DeepCare [30]	✓ (Mode 1)	✓ (Mode 2)	-	-	-	-	P1
T-LSTM [14]	✓ (Short-term only)	-	✓	-	-	-	P1
Time-LSTM [31]	-	✓ (T1, T2)	-	-	-	-	P1
ATTAIN [32]	✓ (Continuous decay)	-	-	-	✓	-	P1
MWTA-LSTM [15]	-	✓	✓	-	-	-	P1
Phased LSTM [18]	-	-	-	-	-	✓	P1
pLSTM [13]	-	-	-	✓	-	-	P2
T-pLSTM [33]	✓ (Short-term)	-	✓	✓	-	-	P1 + P2
BiT-pLSTM [33]	✓ (Short-term)	-	✓	✓	-	-	P1 + P2 (Bidirectional)

**Table 2:** Comparative summary of gate-modifying LSTMs and some alternative architectures for irregular temporal data.

Model	Decay Type	Temporal Mechanism	Computational Complexity	Additional Parameters	Memory Cost	Interpretability	Application Domain	Key Strength	Key Limitation
Standard LSTM [3]	Multiplicative, content-dependent (no explicit ∆t term)	No time-gap mechanism; equidistant step assumption	O(T·d^2^) per sequence (baseline)	None (standard gates only)	O(T·d) activations	Low	General sequence modelling	Gradient stability; well-understood gating	Blind to Δt; underweights long-range dependencies
DeepCare [30]	Logarithmic/parametric	Forget gate scaled by Δt; optional clinical intervention embedding	O(T·d^2^) + minor decay overhead	Decay-scaling weights + diagnosis/intervention embeddings	Slightly higher than LSTM (embedding tables)	Moderate (decay factor interpretable; embeddings less so)	Healthcare/HER (diabetes progression and readmission)	First time-aware LSTM; intervention-conditioned forgetting	Full cell state decays indiscriminately; no subspace split
T-LSTM [14]	Inverse/logarithmic	Subspace decomposition; only the short-term component is discounted	O(T·d^2^) + minor decomposition overhead	Decay-transform weight matrix Wd for short-term subspace	Slightly higher than LSTM	Moderate (short/long split is inspectable)	Healthcare (patient subtyping and clinical time series)	Preserves long-range context; selective short-term decay	Single-step update, with standard forget gate retained
Time-LSTM [31]	Sigmoid-weighted time gate	Dedicated time gate(s) with ∆t; dual gates (T1 and T2) for interest types	O(T·d^2^) with dual-gate overhead	Dual-gate overhead; two extra gate weight matrices (T1 and T2)	Higher than LSTM (extra gates)	Moderate (gates separate short-/long-term interest)	Recommender systems (session-based recommendation)	Architecturally separates short- vs. long-term interest	No retrospective attention; limited explainability
ATTAIN [32]	Continuous time	Attention over all past states weighted by salience and temporal decay	O(T^2^·d) (full attention over history)	Attention/salience weight matrices; decay parameters	High (retains all past cell states)	High (attention weights give attribution)	Healthcare (septic shock progression; HER)	Interpretable attribution map over past clinical events	Higher cost; does not handle multi-frequency noise
MWTA-LSTM [15]	Time and frequency based	Two stacked units: time gate and frequency-augmented forget gate	O(T·d^2^) with added parameterisation overhead	Frequency/last-observation parameterisation weights	Higher than LSTM	Low–moderate	Multivariate ICU/clinical time series (irregular sampling)	Handles elapsed time and sampling frequency jointly	Updates at every arrival
Phased LSTM [18]	Oscillation based (no decay)	Time gate with learnable period, phase, and open ratio	Lower than standard LSTM; sparse updates substantially reduce total update count	Period τ, phase shift s, and open ratio $r_{on}$ per unit	Lower effective runtime memory (sparse updates), despite added parameters	Moderate (learnable rhythms inspectable per unit)	Event-based/asynchronous sensor data; audio	Decouples updates from data arrival; substantially fewer updates than standard LSTM	Fixed oscillation; misses aperiodic events
pLSTM [13]	Power-law	Power-law forget gate	O(T·d^2^) + minor overhead	Power-law exponent parameter(s); ~3/4 the parameters of standard LSTM per reported experiments	Slightly lower than LSTM	Moderate (exponent interpretable as decay rate)	General long-range sequence modelling (copy task, sentiment, and sequential MNIST)	Heavy-tailed decay captures long-range dependencies	No time-gap awareness; equidistant step assumption
T-pLSTM [33]	Power-law and time-scaled subspace	T-LSTM subspace decomposition + pLSTM power-law forget gate	O(T·d^2^) + combined subspace/decay overhead	Subspace decomposition matrices + power-law coefficient p	Higher than LSTM (both parameter sets)	Moderate	Healthcare/EHR (NSCLC tumour size and survival prediction; SEER data)	Only model combining ∆t correction with non-exponential decay	Adds two temporal correction mechanisms concurrently; in the source thesis, performance gains degraded when training data were sparse (short patient sequences)
BiT-pLSTM [33]	Power-law and time-scaled (bidirectional)	Bidirectional T-pLSTM; processes sequences forward and backward	~2× T-pLSTM (forward + backward pass)	T-pLSTM parameters duplicated per direction	~2× T-pLSTM	Moderate	Retrospective clinical sequence analysis (same NSCLC dataset)	Adds bidirectional context	Empirically inconsistent gains over T-pLSTM; in the source thesis, it underperformed T-pLSTM on several survival-month comparisons
Latent ODE [23]	Continuous ODE trajectory	Sequence encoded (e.g., via RNN/ODE-RNN) into a latent initial state; a neural network parameterises dz/dt, and an ODE solver evolves the latent trajectory forward through arbitrary Δt	O(T·NFE·d^2^); NFE = solver function evaluations (variable)	ODE-function network weights, encoder network, and solver tolerance	High (naive backprop); reducible via adjoint method	Low (continuous trajectory and dynamics probed only indirectly)	Irregularly sampled time series (originally physiological/synthetic benchmarks)	Principled continuous-time handling; no discrete step bias	Solver latency; error accumulation on long sequences
Neural CDE [25]	Continuous, data-controlled trajectory	dz/dt = f(z,t)·dX/dt, where X is a continuous interpolation of the observed data acting as a control path	O(T·NFE·d^2^); similar solver-dependent cost to Latent ODE	Vector-field network weights + data interpolation scheme	High; reducible via adjoint method	Low	Irregularly sampled time series with incoming/streaming data	Principled continuous-time handling; no discrete step bias	Solver latency; error accumulation on long sequences
Mamba [35]	Data-dependent linear transition	Discretisation ∆ as dynamic input function; selective history filtering	O (T·d) (sub-quadratic, hardware-aware parallel scan)	Selective SSM projections (Δ, B, and C)	Lower than attention (linear); higher than vanilla LSTM (expanded state)	Low (data-dependent selection is opaque)	Long-context sequence modelling (language, genomics, and audio)	Sub-quadratic training, long-context, and content-selective	Linear time-invariant structure relaxed via a nonlinear, input-dependent selection mechanism (Δ, B, C); no native representation of elapsed time between irregularly sampled observations
mTAN [21]	Not decay-based; continuous-time embedding	Learned continuous-time embedding (generalised positional encoding) + multi-head temporal cross-attention re-represents irregular observations at a fixed reference point	O(T·d) embedding + O(T·R·d) cross-attention; R = reference points	Time-embedding network + multi-head attention weights	Higher than LSTM (stores embeddings for all observations, not a single decayed state)	Moderate (attention weights over reference points are inspectable)	Irregularly sampled multivariate time series (originally clinical, e.g., MIMIC-III)	Learns temporal similarity from data instead of fixed decay kernels; flexible interface to any downstream architecture	Homoscedastic output uncertainty; attention cost scales with number of observations rather than being fixed like a gate
ContiFormer [22]	Not decay-based continuous latent trajectory replaces decay	Neural ODE dynamics embedded inside the transformer’s attention mechanism; queries/keys/values evolve continuously between observations, extending discrete dot-product attention into continuous time	Markedly higher than standard Transformer, quadratic attention plus an ODE solver per attention computation	ODE vector-field network + standard attention projection weights	High (solver-dependent, compounded by quadratic attention memory)	Low (continuous attention dynamics are not directly inspectable)	General irregular time series classification, interpolation, and forecasting benchmarks	Combines continuous-dynamics expressivity of Neural ODEs with the parallelism of attention; breaks the discrete-step assumption of standard Transformers	Substantially higher computation cost than either standard Transformers or the recurrent architectures reviewed here

## Data Availability

No datasets were generated or analysed during the current study.

## Abbreviations

The following abbreviations are used in this manuscript:

AI	Artificial Intelligence
DL	Deep Learning
EHR	Electronic Health Record
GRU	Gated Recurrent Unit
GORU	Gated Orthogonal Recurrent Unit
IOT	Internet of Things
IIOT	Industrial Internet of Things
LSTM	Long Short-Term Memory
MGU	Minimal Gated Unit
MWTA-LSTM	Multi-Way Adaptive Time-Aware LSTM
ODE	Ordinary Differential Equation
SSM	State-Space Model
T-LSTM	Time-Aware LSTM
RNN	Recurrent Neural Network
